# Non-response in a survey of physicians on end-of-life care for the elderly

**DOI:** 10.1186/1756-0500-4-367

**Published:** 2011-09-26

**Authors:** Franziska Kühne, Mareike Behmann, Susanne Bisson, Ulla Walter, Nils Schneider

**Affiliations:** 1 Hannover Medical School, Institute for Epidemiology, Social Medicine and Healthcare Systems Research, Carl-Neuberg-Str.1, 30625 Hannover, Germany

## Abstract

**Background:**

Physicians are quite often surveyed with the aim to investigate their opinions regarding provision and improvement of health care. However, in many cases response rates tend to be rather low. The aim of the study is to reflect methodological aspects regarding survey conduction and to analyse factors that cause physicians to take part in a study on delivering end-of-life care for the elderly.

**Methods:**

N = 4,727 physicians in Lower Saxony, Germany, received a standardised questionnaire on their attitudes about end-of-life care for the elderly. Non-responders were asked to state the reasons for non-participation. Comparison of the sociodemographic characteristics between responders and non-responders, and evaluation of the reasons for non-participation were made.

**Results:**

The response rate to the questionnaire on end-of-life care for the elderly was 40% (n = 1,892). Of the non-responders to the questionnaire, 12.8% (n = 364) stated the reasons for non-participation. Overall, the response rate to the questionnaire varied with specialty and location of the practice: radiotherapists answered significantly more frequently than other categories of physician (e.g. general practitioners) and physicians in rural areas significantly more frequently than their colleagues in urban areas. The reasons most frequently given for non-participation were "Not concerned with the subject" and "No time".

**Conclusions:**

The varying rates of response indicate that the survey was not sufficiently relevant to all groups of physicians, or that the awareness of the topic may be partly underdeveloped.

## Background

In research in the field of palliative medicine, as in other areas, surveys of different groups of people involved are a frequently adopted method of investigating their opinions and their assessments of the situation with regard to the provision of care and to possible ways of improving it. Very often it is physicians who are surveyed, although the response rates often tend to be rather low [1-3].

In a review published in 1991, which covered 219 studies carried out by means of written questionnaires in the United States, the response rates of various groups such as health professionals, patients, relatives and students were compared; the lowest response rate, averaging 54%, was that of physicians [4]. Other studies report response rates of physicians of between 49% and 62% [5-8], and no significant differences in response rates between professional groups [1]. However, it should not be forgotten that surveys producing low response rates are less likely to be published [6].

The response rate is an important criterion for assessing the quality of the data generated by surveys and its suitability as a basis from which generalised conclusions can be drawn [5,9,10]. If only certain subgroups of the sample or population surveyed, e.g. those that have a particular interest in the subject, respond to the survey, distortions in the form of what is known as non-response error may impair the validity of the data and thus of any interpretations derived from it. Where there is a high response, therefore, a low level of non-response error is normally assumed.

A high response rate is usually not the result of an individual factor but of the interplay of a variety of design aspects and features [11,12]. Among others, monetary incentives, short questionnaires, use of reminders and an interesting survey topic were identified as factors increasing the response [2,6,12,13].

### Setting

This study is part of a health services research project on the subject of palliative care for the elderly in Germany, in the course of which the views of physicians from a variety of disciplines with non-hospital-based practices are being investigated with regard to the existing problems of care provision and possible ways of solving them. The overall objective of the research project, in accordance with demands for the further development of palliative medicine [14], is to draw attention to the question as to how palliative care can best be provided for the elderly.

### Objectives and matter investigated

This study investigates which physicians did not take part in the survey on palliative care for the elderly, and for what reasons. In addition, the measures taken to optimise response are examined critically from a methodological point of view. From the findings, recommendations are derived for carrying out studies that involve questionnaires. In particular, answers are sought to the following main questions:

i. Are there differences between participants and non-participants concerning sociodemographic characteristics?

ii. What reasons are given for non-participation?

## Methods

### Study participants and study instrument

4,800 family doctors and specialists working in practices approved by the public health insurance in the State of Lower Saxony, Germany, were included. After the exclusion of physicians who could not be contacted and of duplications [2], the valid sample consisted of 4,727 persons.

The questionnaire used was specially developed for the study, taking into account recommendations on the design of questionnaires [2,12], and was entitled "Health care of elderly people in the last phase of life". The terms "palliative care" or "end-of-life care"were not used in the title, since in our experience physicians in Germany often associate this exclusively with tumour patients or with the terminal stage, whereas the survey was intended to cover a broader range.

The questionnaire consisted of 30 closed and four open questions on the following main topics: number of elderly patients in the last phase of life treated in the physicians' practice; the physicians' assessment of services and cooperation partners available; assessment of the most recent health policy measures regarding palliative and end-of-life care; existing or desirable initial and in-service training; the physician's professional satisfaction and sociodemographic data.

The first version of the questionnaire was subjected to a pre-test using the probing procedure [15], and then revised; this was followed by a second pre-test round and a further revision.

### Survey procedure

All participants included in the sample were sent a questionnaire with a covering letter signed by hand and a data protection declaration, an invoice form for the payment of €20 in appreciation of their efforts, and a stamped address envelope. In the second phase of the survey, which took place four weeks later, a reminder letter and a stamped postcard were added to these documents.

On the postcard the physicians were asked to tick one or more of six stated reasons for non-participation. The intention was to obtain a more detailed characterisation of the group of non-respondents from the distribution of the answers. The standard answer choices offered were *Not concerned with the subject in my everyday work, Not interested in the subject, No time, Generally do not take part in surveys *or *Not approved by the public health insurance*. In addition, there was a category *Other reasons *under which explanations could be given in free text format. Some participants sent a letter or an e-mail to state why they were not taking part in the survey. These answers were included in the analysis together with the postcards; if a physician responded twice, only one response was included in the evaluation.

### Measures used to increase the response rate

To increase the response rate, most common recommendations from the literature [2,12] were implemented in this survey. Table 1 provides a summary of measures recommended in the literature and of the measures realised in this survey.

**Table 1:** Realisation of recommendations for increasing the response rate

	Strategy*	**Recommendation from the literature **[2,12]	Realised in the study	
			Yes	No
**Envelope**		Logo	√	
	P	Postage stamp (if N is small)		Large N
	P	Address personally	√	

**Cover letter**	T	Reliable letter-head/letter-head of the university	√	
	P	Personal form of address	√	
		Explanation of aims of the study	√	
		Information about researchers		On demand
	T	Ensure anonymity	√	
		Name of the researchers/institution	√	
		Telephone number	√	
		Office hours of the researchers		X
	P	Hand-written signature	√	
		Not longer than 1 page	√	
		Clarify importance of participation	√	
		Brief information about results		On demand

**Questionnaire**		A4, white, tacked	√	
	C	Short, not more than 16 pages	√	
	T	Easily readable and understandable/blocks of questions	√	
		No sociodemographic/precarious questions at the beginning	√	
		Preferably closed response format	√	
		Preferably no filter questions	√	
		Consider rules to formulate questions (e.g. no double negative)	√	
		Front page: title, institution, address, contact	√	
		Directions on how to fill in the questionnaire		X
		At the end: 1/2 page for comments	^1^/_4 _page	

**Privacy policy**		How are data handled?		X
		Ensure anonymity	√	
		Ensure no disclosure of data		X
		Data protection officer involved	√	

**Envelope for reply**		Return free of charge	√	

**Announcement**	C	Only if no intensive follow-up	√	

**Salience**	T	Interesting topic	√	

**Follow-up**	F	First dispatch, thank-you note/reminder, second dispatch of questionnaire	√	

**Recognised authorities**		Here: newspaper article, cover letter of National Medical Council	√	

**Material incentives**	C	Monetary: no delayed incentives		Delayed fixed amount
	C	Non-monetary (e.g. brochure)		X

*Strategy: P...Personalisation; C...Commitment; T...Trust in researcher; F...Follow-up

### Analysis

The data was analysed descriptively, using the statistical software SPSS 16.0. The chi-square test was used to statistically evaluate differences between groups; the level of significance was set at *P *< .05. The free text answers were repeatedly read and inductively categorised according to main topics.

### Ethics

In keeping with usual practice at Hannover Medical School concerning this kind of research, the chair of the local ethics committee was consulted prior to the start of the study. Formal approval by the Ethics Committee was not necessary because no patient data was collected and no experiments were performed on human beings. All participants were informed of the background, process and objectives of the study and were able at any time to end their participation without giving any reasons. All data were anonymously treated and exclusively used for scientific purposes.

## Results

### Description of sample

74.1% of the physicians included in the sample were in the age group 40-59 years. 68.9% were men. Most physicians (68.0%) worked as family doctors (general practitioners or internists). Around 38.4% of the practices were located in medium-sized towns (20,000-100,000 inhabitants).

**Figure 1:**
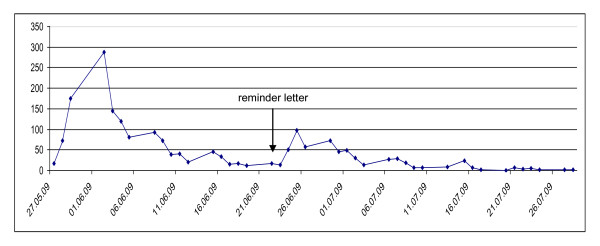
**Number of questionnaires returned during the study period**.

The response rate to the questionnaire on end-of-life care for the elderly was 40.0% (n = 1,892 out of n = 4,727). Figure 1 shows how the returns were spread over time during the study period. Of the non-respondents (n = 2,835), 12.8% (n = 364) sent in a postcard or an e-mail.

### Differences between participants and non-participants

The response to the survey varied significantly according to sex, medical discipline and location of practice (Table 2): 43.3% (n = 581) of women and 37.9% (n = 1268) of men answered (*P *= .001). With respect to medical disciplines, 52.4% (n = 22) of radiotherapists responded compared to 32.7% (n = 106) of neurologists (*P *= .003). Concerning the practice location, the response rate ranged from 61.1% (n = 280) for physicians practicing in a community with less than 5,000 inhabitants to 31.5% (n = 134) for physicians practicing in major cities (*P *< 0.001).

**Table 2:** Group differences between survey responders and non-responders

Sociodemographic characteristics (*P*)*		Survey responders		Survey nonresponders		Totals
		
		%	n	%	n	n
Sex (.001)	Male	37.9	1268	62.1	2074	3342
	Female	43.3	581	56.7	761	1342

Medical disciplines (.003)	General practice	39.8	897	60.2	1357	2254
	Internal medicine	40.6	423	59.4	619	1042
	Gynaecology	34.1	172	65.9	332	504
	Neurology	32.7	106	67.3	218	324
	Urology	37.2	102	62.8	172	274
	Psychiatry/Psychotherapy	45.6	98	54.4	117	215
	Radiotherapy	52.4	22	47.6	20	44

Practice location (<.001)	Other community (< 5,000 inhabitants)	61.1	280	38.9	178	458
	Small town (5,000-20,000 inhabitants)	37.0	374	63.0	638	1012
	Medium-sized town (>20,000-100,000 inhabitants)	36.0	670	64.0	1191	1861
	Large town (>100,000-500,000 inhabitants)	41.8	383	58.2	534	917
	Major city (> 500,000 inhabitants)	31.5	134	68.5	291	425

*chi-square test

### Reasons for non-participation

Table 3 collates the reasons for non-participation that the physicians stated on the reply postcards, giving multiple answers if they wished. N = 364 persons gave a total of 424 answers (max. 3) by postcard, letter or e-mail, making an average of 1.2 stated reasons per respondent.

**Table 3:** Reasons for non-participation (n = 364 answers)

Reasons for non-participation (multiple answers possible)	n	%
Not concerned with the subject in my everyday work	154	42.3
No time	93	25.5
Generally no participation in surveys	55	15.1
(At present) Not accredited	47	12.9
Not interested in the subject	6	1.6

Other reasons	69	19.0

The most frequently given reason (42.3%; n = 154) was *Not concerned with the subject in my everyday work*. Some of those who answered by postcard (n = 42) added in free text that they had scarcely any patients, or none at all, in the category concerned, or that they possessed health insurance approval only in respect of certain specific conditions. The following are examples of the reasons given for missing participation in the survey: dialysis practice, antenatal diagnostics practice, paediatric osteopathy, practice focusing on traditional Chinese medicine, practice working only in the field of psychotherapy or psychoanalysis or, for example, of sexual medicine or of psycho-oncology.

The second most frequent answer given, at 25.5% (n = 93), was having *No time*. The third most frequently given answer, by 19.0% (n = 69) was *Other reasons*: 38 of these 69 physicians described this in more detail; the most frequent *Other reasons *were that the physicians had *retired*, had *closed their practices *(n = 16) or had *moved away *(n = 9). Other reasons for not answering were *Generally do not take part in surveys *(15.1%; n = 55) and *Not approved by the public health insurance *(12.9%; n = 47). 1.6% (n = 6) of the respondents stated that they were *Not interested in the subject*.

Of the physicians specialising in gynaecology, 75.0% (n = 42) stated that they were *not concerned with the subject*, as did 85.2% (n = 23) of psychiatrists/psychotherapists. By contrast, only 20.3% (n = 27) of general practitioners said they were *not concerned with the subject *(*P *< .001).

40.6% (n = 13) of the neurologists and 31.6% (n = 42) of the general practitioners did not take part because of *lack of time*; among gynaecologists the figure was 12.5% (n = 7) and among psychiatrists/psychotherapists 7.4% (n = 2) (*P *= .011).

## Discussion

### Response rate

There has been an increasing amount of research in recent years focusing on palliative and end-of-life care. Surveys carried out in Britain and America, throughout Europe and in German-speaking countries [e.g. [16-22]] have all produced large variations in the rates of response to surveys relating to palliative medicine, which range from around 20% to around 60%. These differences in response rates are largely due to aspects of the methodology and the design of the questionnaires [12,13]. In our survey, the response rate of 40% was in the middle range as compared with the figures for other studies reported in the literature.

Amongst other things, more (quasi-)experimental studies and systematic reviews or meta-analyses would be desirable to systematically analyse methodological reasons for variations in response rates. In addition, few studies have so far been published which combine a variety of survey modes in order to enhance the response rate [23].

According to the literature [15,24], in this study a financial incentive ("expense allowance") of €20 was paid, this being assumed to be appropriate compensation, in the light of the levels of remuneration prevailing in Germany for various individual services performed in doctors' surgeries. From a number of responses made by telephone or by post, we did gain the impression that our decision to make a payment was perceived as an expression of appreciation and motivated physicians to take part.

### Respresentativity

The fact that around 70% of both the participants and the non-participants in this study were men corresponds to the distribution of physicians in practice outside hospitals in the study region, where 66.1% of the physicians working in their own surgeries or otherwise outside hospitals were men in 2008 [25]. The fact that most participants were general practitioners, followed by specialists in internal medicine, also corresponds to the distribution of physicians in the State of Lower Saxony. This suggests that, overall, the doctors who answered our questionnaire can be considered as largely representative of the physicians in the study region.

### Participation rates of different medical disciplines

According to the literature, an interesting survey topic is a factor increasing the response [15]. Taking into account that end-of-life care for the elderly is a highly relevant topic for general practice [26,27], we expected an over-average participation rate of general practitioners. However, their participation rate lay in the middle range as between the different medical disciplines. This no more than average rate of participation by GPs may partly be explained by lack of time, as they are one of those groups whose members stated significantly more frequently that their non-participation was due to lack of time.

Participation was highest among radiotherapists and lowest among neurologists. The fact that radiotherapists were more likely to take part may be due to the fact that those radiotherapists who answered have more elderly people in the last phase of life as patients than neurologists do. However, it should also be noted that the group of radiotherapists comprised a mere 22 persons.

### Practice location

Physicians whose practices are located in communities with less than 5,000 inhabitants were significantly more likely to take part. Another study reports no difference in the rate of participation in relation to differences of region and urbanicity [11]. Our differing findings may be due to the situation that physicians in smaller places have closer relationships with their patients, and that the salience of the subject of the survey and their willingness to take part were higher as a result.

### Obstacles to participation arising out of thematic and time factors

Only 12.8% (n = 364) of the non-respondents gave their reasons for non-participating which is a major limitation. Therefore, conclusions should be made with caution. However, the findings provide some insights into reasons for non-participation.

The most frequently given reason was that the physician was *Not concerned with the subject; *the second most frequent was lack of time. As far as the reasons are concerned, these findings correspond to those stated in a study in which general practitioners and specialists were questioned on the subject of euthanasia and physician-assisted suicide, although the frequencies differ [17]. The reasons for non-participation most frequently stated in that study were lack of time (42%) and not being concerned with the target population and the subject matter (29%).

Among *Other reasons *stated for non-participation was working in a different field (e.g. in paediatrics, or only in psychotherapy/psychoanalysis). Some physicians were no longer active in their profession. The overall sample was based on a directory provided by the Lower Saxony Chamber of Physicians, which was probably not completely up to date contrary to the statement of the Chamber of Physicians. In future studies, therefore, the data source for determining the overall sample should be examined with regard to its precise update status.

Those physicians who are not strongly involved in delivering end-of-life care for the elderly could have tended to exercise self-selection, and so were not so frequently represented among the responders. Thus gynaecologists and psychiatrists/psychotherapists stated significantly more frequently than general practitioners that they were not concerned with the subject. This is plausible and correlates with another study on the subject of medical end-of-life decisions, in which non-respondents were found to have significantly fewer patients in the terminal stage than respondents [28].

## Conclusions

The overall response rate to the questionnaire was satisfactory, with good representativity of the physicians practicing in the study region. The methodological approaches to optimise the response have proved successful. However, the varying rates of response indicate that the survey was not sufficiently relevant to all groups of physicians, or that the awareness of the topic may be partly underdeveloped.

## Competing interests

The authors declare that they have no competing interests.

## Authors' contributions

FK, MB and NS developed the questionnaire, carried out the study and interpreted the data. FK, MB and SB carried out the analysis. FK and NS drafted the manuscript. UW helped to draft the manuscript. All authors read and approved the final manuscript.
